# Molecular and phenotypic characteristics of 15q24 microdeletion in pediatric patients with developmental disorders

**DOI:** 10.1186/s13039-021-00574-x

**Published:** 2021-12-18

**Authors:** Yuanyuan Zhang, Xiaoliang Liu, Haiming Gao, Wanting Cui, Bijun Zhang, Yanyan Zhao

**Affiliations:** Department of Clinical Genetics, Shengjing Hospital of China Medical University, Shenyang, 110004 Liaoning China

**Keywords:** 15q24 microdeletion, Developmental delay, MLPA

## Abstract

Chromosome 15q24 microdeletion is a rare genetic disorder characterized by development delay, facial dysmorphism, congenital malformations, and occasional autism spectrum disorder (ASD). In this study, we identified five cases of 15q24 microdeletion using multiplex ligation-dependent probe amplification (MLPA) technology in a cohort of patients with developmental delay and/or intellectual disability. Two of these five cases had deletions that overlapped with the previously defined 1.1 Mb region observed in most reported cases. Two cases had smaller deletions (< 0.57 Mb) in the 15q24.1 low copy repeat (LCR) B-C region. They presented significant neurobehavioral features, suggesting that this smaller interval is critical for core phenotypes of 15q24 microdeletion syndrome. One case had minimal homozygous deletion of less than 0.11 Mb in the 15q24.1 LCR B-C region, which contained *CYP1A1* (*cytochrome P450 family 1 subfamily A member 1*) and *EDC3* (*enhancer of mRNA decapping 3*) genes, resulting in poor immunity, severe laryngeal stridor, and lower limbs swelling. This study provides additional evidence of 15q24 microdeletion syndrome with genetic and clinical findings. The results will be of significance to pediatricians in their daily practice.

**Supplementary Information:**

The online version contains supplementary material available at 10.1186/s13039-021-00574-x.

## Introduction

The rare genetic disorder 15q24 microdeletion is caused by nonallelic homologous recombination (NAHR) between low copy repeats (LCRs) in the chromosome 15 band q24 region; the overall incidence approaches 1 in 42,000 in the general population [1]. Among subjects with intellectual disability and various congenital defects, the prevalence is approximately 1 in 1751 [2]. To the best of our knowledge, more than 60 patients with 15q24 deletions have been described in the literature. The majority of 15q24 microdeletions identified to date involve a 1.1 Mb region between 72.2 and 73.3 Mb of the reference genome (NCBI36/hg18) [3]. A few cases were reported to have atypical deletions, involving only a part or none of the proposed 1.1 Mb critical region, but still appeared to be dysfunctional [4]. The clinical spectrum of 15q24 microdeletion syndrome is variable. Most patients showed developmental delay, intellectual disability, distinctive facial features, and significant speech delay and hypotonia in childhood; some patients manifest eye abnormalities, frequently strabismus, digital anomalies, and dysplastic ears; individual patients also have joint laxity, hearing loss, brain abnormalities, and urogenital malformation symptoms [3].

**Fig. 1:**
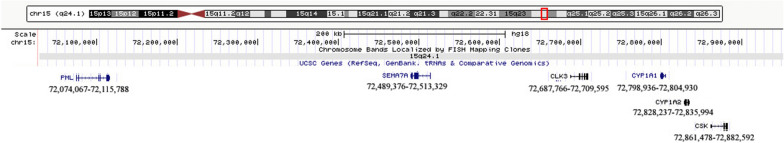
Region of 15q24.1 from the UCSC Genome Browser based on the Genome Reference Consortium Human Genome (NCBI36/hg18). The positions of genes associated with MLPA P245 and P371 probe mixes were labeled

The 15q24 microdeletion can be detected by molecular methods that determine the copy number of sequences within the deleted region. In China, multiplex ligation-dependent probe amplification (MLPA) is a rapid and cost effective method for detection of targeted chromosome copy number variations (CNVs). The process from DNA extraction to capillary electrophoresis can be completed within 24 h at a cost of 100 to 200 RMB. However, the detection rate of potential pathogenic variation achieved by MLPA is lower than that by microarray. The MLPA P245 probe mix is designed to screen common microdeletion and microduplication syndromes associated with developmental disorders. It contains two probes for the chromosome 15q24 (CYP1A1-2 and SEMA7A-11) region. The MLPA P371 probe mix is specific to this region, including nine probes for the *PML* [*PML nuclear body scaffold*] (2), *SEMA7A* [*semaphorin* *7A*] (2), *CLK3* [*CDC like kinase 3*] (1), *CYP1A1* [*cytochrome P450 family 1 subfamily A member 1*] (2), *CYP1A2* [*cytochrome P450 family 1 subfamily A member 2*] (1) and *CSK* [*C-terminal Src kinase*] (1) genes. The MLPA probe names and their exact locations in the genome are listed in Additional file 1: Table S1, and the locations of related genes are shown in Fig. 1. Here, using MLPA assay, five pediatric patients with developmental disorders were identified to have 15q24 microdeletions, and the deletion sizes were different. Two carried microdeletions of < 0.57 Mb in the LCR B-C region and showed significant neurobehavioral features. Our results help to delineate the critical region for core phenotypes of 15q24 deletion syndrome.

## Methods

### Patients and samples

A cohort of 7077 cases was enrolled in this study. They were recruited from outpatient department of pediatrics and clinical genetics of Shengjing Hospital of China Medical University. Their ages ranged from 30 days to 12 years. Most presented with developmental disorders (such as motor delay, speech or language delay, and intellectual disability.) Other manifestations were also observed, including congenital malformation, craniofacial abnormalities, and behavioral problems. This study was approved by the Ethics Committee of Shengjing Hospital of China Medical University. Considering these cases were all under the age of 16, written informed consents to participate were obtained from their parents or legal guardians.

### Genomic DNA preparation

Automatic nucleic acid extractor (Allsheng Auto-Pure 32A) was applied to extract genomic DNA from the whole peripheral blood, using a UPure Blood DNA Extraction Kit (M2002-A32; BioBase Technologies Co., LTD). DNA concentration was quantitated using a NanoDrop 1000 (Thermo Scientific, USA). A concentration of 10–50 ng/μL DNA was prepared for MLPA assay.

### Multiplex ligation-dependent probe amplification (MLPA) assay

MLPA assay was carried out in accordance with the manufacturer’s instructions. The SALSA P245 probe mix (MRC-Holland, Netherlands) was used to screen for microdeletion/microduplication syndromes as previously described [5]. The SALSA P371 probe mix (MRC-Holland, Netherlands), which encompasses more probes specific for the chromosome 15q24 region, was used to confirm the deletion and infer the deletion size. The amplification products were loaded to ABI 3730 Genetic Analyzer (Applied Biosystems, USA) for capillary electrophoresis. The raw data were generated by the GeneMapper software (Applied Biosystems, USA), and analyzed using the Coffalyser Net software (MRC-Holland, Netherlands).

## Results

### Identification of 15q24 microdeletion

**Fig. 2:**
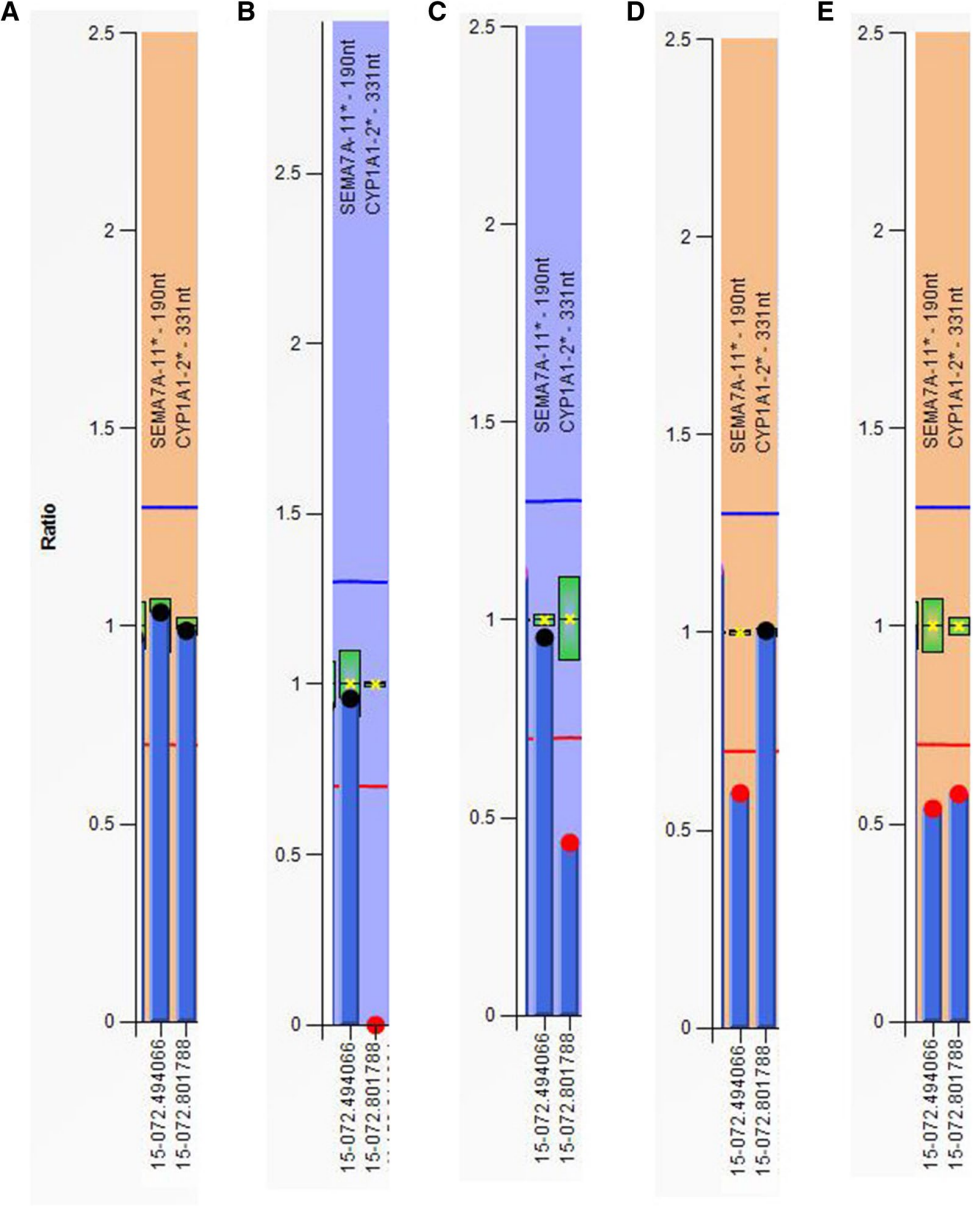
Columns of MLPA P245 results. X-axis represents MLPA probes. Y-axis represents probe dosage ratio. The blue line indicates probe dosage ratio of 1.35, and ratio above this line represents duplication. The red line indicates probe dosage ratio of 0.65, and ratio below this line represents deletion. Ratios between 0.85 and 1.15 are considered as normal. **A** A control with normal copy probes. **B** Case 1 carries CYP1A1-2 homozygous deletion. **C** Mother of case 1 carries CYP1A1-2 heterozygous deletion. **D** Case 3 carries SEMA7A-11 heterozygous deletion. **E** Case 4 carries CYP1A1-2 and SEMA7A-11 heterozygous deletion. MLPA: Multiplex ligation-dependent probe amplification

**Fig. 3:**
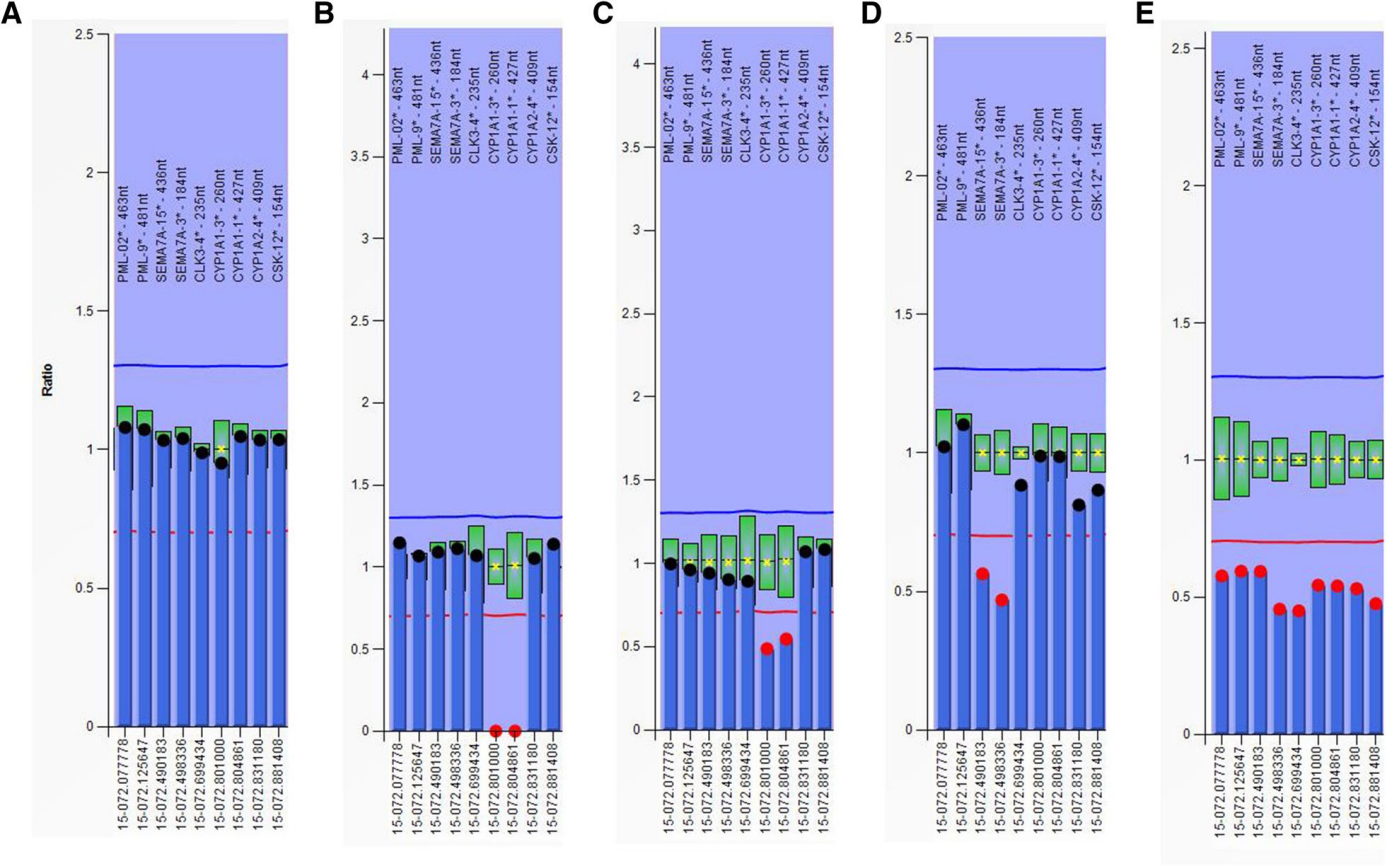
Columns of MLPA P371 results. X-axis represents MLPA probes. Y-axis represents probe dosage ratio. The blue line indicates probe dosage ratio of 1.35, and ratio above this line represents duplication. The red line indicates probe dosage ratio of 0.65, and ratio below this line represents deletion. Ratios between 0.85 and 1.15 are considered as normal. **A** A control with normal copy probes. **B** Case 1 carries CYP1A1-1 and CYP1A1-3 homozygous deletion. **C** Mother of case 1 carries CYP1A1-1 and CYP1A1-3 heterozygous deletion. **D** Case 3 carries SEMA7A-3 and SEMA7A-15 heterozygous deletion. **E** Case 4 carries heterozygous deletion from PML to CSK probes. MLPA: Multiplex ligation-dependent probe amplification

Of the 7077 cases examined by MLPA P245 assay, five were found to have genomic imbalance at 15q24, with a prevalence of 1 in 1429. We named these five cases numbers 1 to 5. Case 1 was 55 days old; cases 2, 3, 4, and 5 were 2–3 years old. The MLPA P245 results showed that case 1 had homozygous deletion at the CYP1A1-2 probe; cases 2 and 3 had heterozygous deletions at the SEMA7A-11 probe; cases 4 and 5 had heterozygous deletion at both the CYP1A1-2 and SEMA7A-11 probes (Fig. 2). All five cases were further confirmed to have deletions at 15q24 by MLPA P371 assay, including those with single probe abnormalities by P245 assay, which ruled out the possibility of probe binding sequence mutation. In detail, cases 4 and 5 showed heterozygous deletion at all nine probes; cases 2 and 3 had heterozygous deletions at two probes of the *SEMA7A* gene; case 1 had homozygous deletions at two probes of the *CYP1A1* gene (Fig. 3). Parental analysis revealed that the deletions were de novo in cases 2, 3, 4, and 5. The deletion of Case 1 was inherited from her mother, who had heterozygous deletions at the same probes; her father was normal. Detailed results of MLPA tests are shown in Table 1.

**Table 1:** Clinical information for the cases with 15q24 deletions

Case number	Sex	Age	Variation	Oringin	Abnormal probes		Clinical manifestations
					P245	P371	
1	Female	55 days	15q24 homozygous deletion	Matermal	CYP1A1-2	CYP1A1-1 CYP1A1-3	Laryngeal stridor, Lower limbs swelling
Mother of case 1	Female	32 years	15q24 heterozygous deletion	Unknown	CYP1A1-2	CYP1A1-1 CYP1A1-3	Normal
2	Male	2 years	15q24 heterozygous deletion	De novo	SEMA7A-11	SEMA7A-15 SEMA7A-3	Speech or language difficulty, little vocabulary Motor delay, walked at 18 months Poor response when called Absence of pointing Social interaction impairment Hyperactivity DQ: 72 CARS: 19
3	Female	3 years	15q24 heterozygous deletion	De novo	SEMA7A-11	SEMA7A-15 SEMA7A-3	Speech or language difficulty, nonverbal vocabulary Abnormal behavior, listen to songs repetitively, jump aimlessly Poor response when called Absence of pointing Social interaction impairment No eye contact Unable to follow instruction Hyperactivity Timid DQ: 53 CARS: 30
4	Female	3 years	15q24 heterozygous deletion	De novo	CYP1A1-2 SEMA7A-11	PML-2 PML-9 SEMA7A-15 SEMA7A-3 CLK3-4 CYP1A1-3 CYP1A1-1 CYP1A2-4 CSK-12	Speech or language difficulty, unable to speak a complete sentence Motor delay, walked at 19 months Abnormal behavior, Unable to follow instruction DQ: 62 CARS: 27
5	Female	3 years	15q24 heterozygous deletion	De novo	CYP1A1-2 SEMA7A-11	PML-2 PML-9 SEMA7A-15 SEMA7A-3 CLK3-4 CYP1A1-3 CYP1A1-1 CYP1A2-4 CSK-12	Speech or language difficulty, speaking single word Motor delay, walked at 19 months Poor understanding Special facial appearance, wide eye distance DQ: 81 CARS: 22

*DQ* developmental quotient, *CARS* childhood autism rating scale

### Clinical phenotypes of cases with 15q24 microdeletion

Cases 2, 3, 4, and 5 had varying degrees of speech or language difficulties from being unable to speak a complete sentence to nonverbal vocabulary. Besides, they manifested motor delay, intellectual disability, and neurobehavioral disorders, including impairment in social interaction, poor response when called, and absence of pointing. Individual children also showed special facial appearance, hyperactivity, and abnormal behaviors. Specifically, case 5 had wide eye distance and strabismus; cases 2 and 3 were hyperactive; case 3 liked to listen to songs repetitively and jumped aimlessly. These children were tested using the childhood autism rating scale (CARS) [6] to assess the existence and severity of autism. Case 3 had a score of 30, meeting the critical value (scores greater than or equal to 30 denote autism, with a score between 30 and 36 considered as mild); cases 2, 4, and 5 had scores of < 30. Case 1, a baby girl with CYP1A1 probes homozygous deletion, presented with severe laryngeal stridor and lower limb swelling. She had obvious three concave sign, with a respiration rate of 35 times per minute and a heart rate of 130 times per minute. She had suffered from pneumonia and pyemia before. She passed away shortly after being admitted to the pediatric emergency ward. However, her mother, who also carried CYP1A1 probes heterozygous deletion presented as normal. Clinical information and phenotypes are shown in Table 1.

## Discussion

Chromosome 15 band q24 is a complex genomic region with five LCRs, referred to as A, B, C, D, and E. These LCRs can promote NAHR at meiosis, resulting in deletion of the interval sequence. Most deletions are between 1.7 to 6.1 Mb in size, encompassing a small critical region spanning 1.1 Mb in 15q24.1 LCR B-C [1, 3]. MLPA P245 and P371 probe mixes have 11 probes scattered in this small region. Cases 4 and 5 had heterozygous deletions at all 11 probes, indicating that the deletions covered this 1.1 Mb interval. Cases 2 and 3 had heterozygous deletion at probes of only the *SEMA7A* gene, suggesting that the fragments involved were between the locations of the *PML* (72.11 Mb) and *CLK3* (72.68 Mb) genes and that the deletion sizes were less than 0.57 Mb. Cases 2 and 3 both had obvious developmental delays and neurobehavioral features, illustrating that the haploinsufficiency of this tiny fragment could lead to the main manifestations of 15q24 deletion syndrome. This interval contains the *STRA6* (*signaling receptor and transporter of retinol STRA6*), *CCDC33* (*coiled-coil domain containing 33*), *CYP11A1* (*cytochrome P450 family 11 subfamily A member 1*), *SEMA7A*, and *UBL7* (*ubiquitin-like 7*) genes, which may contribute to the corresponding phenotypes of the syndrome. SEMA7A is expressed in neuron. It enhances central and peripheral axon growth, and is required for proper axon tract formation during embryonic development [7]. STRA6 is involved in cellular uptake of vitamin A [8]. Haploinsufficiency of STRA6 may cause a group of congenital malformations, including microphthalmia, cardiovascular malformations, diaphragmatic hernia, and mental retardation [9]. The function of *CCDC33* gene is rarely reported. A recent study demonstrated CCDC33 may have potential roles in immunity through interacting at a distance with and modulating *ISLR2* (*immunoglobulin superfamily containing leucine rich repeat 2*) gene expression [10]. CYP11A1 encodes a mitochondrial cholesterol side-chain cleavage enzyme that catalyzes side-chain hydroxylation and cleavage of cholesterol to pregnenolone, the precursor of most steroid hormones [11]. The haploinsufficiency of CYP11A1 may contribute to the genital abnormalities in patients with 15q24 deletion syndrome [12]. Little is known about UBL7 (BMSC-UbP), which possesses a ubiquitin-associated domain [13]. It is widely expressed and may play a role by regulating substrate protein expression through ubiquitination modification.

The 15q24 deletion occurs de novo in most reported cases. To the best of our knowledge, only one study described three patients in the same family [14]. In this study, case 1 and her mother had deletions at the same probes, suggesting that her mother’s deletion was delivered to her. However, case 1 had homozygous deletion, whereas her mother had heterozygous deletion. This 55 days old baby manifested severe laryngeal stridor and lower limb swelling. We did not observe any developmental problems because she was too young, but she had suffered from pneumonia and pyemia before, suggesting that her immunity was poor. Unfortunately, she passed away shortly after being admitted to the pediatric emergency ward. However, her mother, who carried heterozygous deletion, was asymptomatic and gave birth to a healthy baby 1 year later, indicating incomplete penetrance of this mutation and that the consequence of homozygous deletion is more serious. Combined with the results of P245 and P371 assays, we concluded that the deletion was small and between the locations of the *CLK3* (72.71 Mb) and *CYP1A2* (72.82 Mb) genes, with a deletion size < 0.11 Mb. Interestingly, we applied next generation sequencing (NGS) based CNV-seq to further determine this tiny deletion—this approach can detect structural abnormalities larger than 100 kb—but did not get a positive result (data not shown). This suggests that the deletion size is actually smaller than 0.1 Mb and exceeds the resolution of CNV-seq. We also tested the sample with the MS-MLPA P028 assay, which was used to analyze CpG island methylation of the 15q11 region. The negative result (data not shown) ruled out the possibilities of entire chromosome 15 uniparental disomy (UPD) and Prader-Willi syndrome (deletion or UPD). We speculated that the homozygous deletion might be due to segmental UPD or paternal loss of this tiny fragment. We wanted to use SNP array to confirm it, but no DNA sample left for us to do this. There are two genes in this interval, *EDC3* (*enhancer of mRNA decapping 3*) and *CYP1A1*. EDC3 is associated with mRNA degradation [15]. Mutations in EDC3 have been identified in autosomal recessive intellectual disability, indicating its crucial role in neurodevelopment [16]. CYP1A1 mediates the metabolism of fatty acids, steroid hormones, vitamins, and drugs. It can metabolize some polycyclic aromatic hydrocarbons (PAHs) to carcinogenic intermediates [17], and is important in defining the efficacy and toxicity/carcinogenicity of drugs and foreign compounds [18].

Genomic CNV is a risk factor for autism spectrum disorder (ASD). Certain CNVs, such as 22q13 deletion, 16p11.2 deletion/duplication, 22q11.2 deletion/duplication, and 15q24 duplication have been found in patients with ASD [19]. ASD was also reported to be an additional phenotype in 15q24 deletion syndrome [20, 21]. Recently, a study observed a critical interval of 0.65 Mb in the 15q24 LCR A-B region, which might contribute to ASD [22]. In our study, case 3, who had a deletion of < 0.57 Mb in the 15q24.1 LCR B-C region, manifested ASD features, including nonverbal communication, social interaction impairment, restricted and repetitive behaviors, hyperactivity, no eye contact, and poor response when called. According to CARS, she had a score of 30 and was diagnosed with mild ASD. She was referred to pediatric neurology for further evaluation and treatment. The aforementioned *SEMA7A* gene plays a critical role in neurodevelopment. A recent study identified SEMA7A as a critical cue that restricts serotonergic innervation in the spinal cord [23]. In PTZ-kindled epileptic rats, Sema7A was upregulated in the epileptic brain and played a potential role in the regulation of seizure activity [24]. *SEMA5A* (*semaphorin 5A*), another gene of the semaphorin family, was identified to be an ASD-associated gene, and there was evidence of decreased expression of this gene in patients with autism [25, 26]. However, the relationship between SEMA7A and ASD is not clear. Further investigation is required to determine the contribution of SEMA7A to the complex etiology of ASD.

In conclusion, this study revealed the prevalence of chromosome 15q24 microdeletion in Chinese pediatric patients with developmental delay and/or intellectual disability. We present additional evidence of 15q24 microdeletion syndrome with genetic and clinical findings. We identified two smaller deletions (< 0.57 Mb and < 0.11 Mb) within the 15q24.1 LCR B-C region that have not been reported in previous data, and delineated a critical region for core phenotypes in 15q24 microdeletion syndrome. Our results will increase awareness of these disruptive mutations among pediatricians and provide meaningful information for their daily practice.

## Supplementary Information

**Additional file 1.** MLPA probes for chromosome 15q24.

## Data Availability

The datasets generated and/or analyzed during the current study are available in the figshare repository (https://figshare.com/articles/figure/Untitled_Item/14832789).

## Abbreviations

ACGH: Array comparative genomic hybridization
ASD: Autism spectrum disorder
CARS: Childhood autism rating scale
CCDC33: Coiled-coil domain containing 33
CLK3: CDC like kinase 3
CNV: Copy number variation
CSK: C-terminal Src kinase
CYP1A1: Cytochrome P450 family 1 subfamily A member 1
CYP1A2: Cytochrome P450 family 1 subfamily A member 2
CYP11A1: Cytochrome P450 family 11 subfamily A member 1
EDC3: Enhancer of mRNA decapping 3
ISLR2: Immunoglobulin superfamily containing leucine rich repeat 2
LCR: Low copy repeat
LOH: Loss of heterozygosis
MLPA: Multiplex ligation-dependent probe amplification
NAHR: Nonallelic homologous recombination
NGS: Next generation sequencing
PAHs: Polycyclic aromatic hydrocarbons
PML: PML nuclear body scaffold
SEMA5A: Semaphorin 5A
SEMA7A: Semaphorin 7A
STRA6: Signaling receptor and transporter of retinol STRA6
UBL7: Ubiquitin-like 7
UPD: Uniparental disomy
