# Supplementary material for: Molecular and phenotypic characteristics of 15q24 microdeletion in pediatric patients with developmental disorders
Source: Mol Cytogenet. 2021 Dec 18;14:57. doi: 10.1186/s13039-021-00574-x (PMC8684056; doi:10.1186/s13039-021-00574-x)
Supplement: Supplementary file 1 — Additional file 1. MLPA probes for chromosome 15q24. [file 13039_2021_574_MOESM1_ESM.doc]

MLPA probes for chromosome 15q24

| Kit | Probes | Genes | Locations* |
| --- | --- | --- | --- |
| P371 | PML-2 | PML | 15-072.077778 |
| P371 | PML-9 | PML | 15-072.125647 |
| P371 | SEMA7A-15 | SEMA7A | 15-072.490183 |
| P245 | SEMA7A-11 | SEMA7A | 15-072.495259 |
| P371 | SEMA7A-3 | SEMA7A | 15-072.498336 |
| P371 | CLK3-4 | CLK3 | 15-072.699434 |
| P371 | CYP1A1-3 | CYP1A1 | 15-072.801000 |
| P245 | CYP1A1-2 | CYP1A1 | 15-072.801788 |
| P371 | CYP1A1-1 | CYP1A1 | 15-072.804861 |
| P371 | CYP1A2-4 | CYP1A2 | 15-072.831180 |
| P371 | CSK-12 | CSK | 15-072.881408 |

*Locations are based on the Genome Reference Consortium Human Genome (NCBI36/hg18).
